# Monthly Digest

**Published:** 1896-10

**Authors:** J. M. Walker


### MONTHLY DIGEST.

Prepared for the DENTAL REGISTER by MRS. J. M. WALKER.

### PROSTHETICS.

The *actual* and the *ideal* is thus portrayed editorially in the consideration of *Specialists in Dentistry.** "We have many very capable specialists in the mere mechanical details. They have attained the perfection of the jeweler's art, but they can go no further. * * The prosthetic specialist, instead of being merely the skillful mechanic, should make the facial character-

*International, April, 1895.

istics a study, have some knowledge of temperaments, study the rules of articulation, in a word, combine in his work all that is meant by the word artistic. He should be given the entire management of the insertion of artificial dentures, and thus bring it to such perfection that 'art will conceal art,' and not, as now, make it the most prominent feature of the work. In this way only, it seems to us, can this important specialty receive the attention it must ever demand."

In a series of five articles entitled

PORCELAIN DENTAL ART,*

DR. W. A. CAPON considers the advantages offered by porcelain work in overcoming deformities and defects in an artistic and acceptable manner, the work proving reliable and satisfactory under the most discouraging conditions for fillings, crowns and bridges.

The porcelain inlay, properly made and adjusted, is the least noticeable and the most artistic method of restoring the anterior teeth where large gold fillings are objectionably conspicuous, and the work long and tedious. This work is espeeially indicated in labial and large proximal cavities and contoured tips, broken centrals and losses by excessive abrasion.

By means of porcelain crowns the most difficult cases can be overcome, as the teeth can be carved to fit any peculiarity. They may be pin crowns, with or without collars, crowns with tubes to fit over posts screwed into the canals, or jacket crowns for teeth with living pulps. This crown can also be used where the roots, for any reason, are useless for the retention of pin crowns.

Illustrations in the March and April issues (*Items of Interest*), show the almost unlimited range of usefulness of the jacket crown in the restoration of the most difficult cases. All porcelain bridge-work requires a thorough knowledge of porcelain furnaces, the oxyhydrogen blow-pipe, etc. Its principal advantages lie in its strength, cleanliness, natural appearance, from the entire absence of gold, and also the low cost of production,

* Items of interests, Jan.-May, 1895.

often a mere fraction of the cost of a gold and porcelain section. It emerges from the furnace in a completely-finished condition, saving the hours of grinding and finishing required with gold and porcelain work.

### GLASS INLAYS.

DR WM. TRUEMAN* gives some interesting paragraphs quoted from an old English work published in 1837, describing glass inlays for use in "cavities in the front of a cutting tooth," also caps of gold, platina or palladium, stamped up to fit, the fronts of the caps to be glazed, to be worn when the teeth are very much decayed, discolored, or have the enamel much injured or disfigured.

### PORCELAIN WORK IN DENTISTRY.

DR. G. W. SCHWARTZ, in a paper read before the Odontographic Society, of Chicago,† reviews the use of porcelain work in dentistry, dating from its practical introduction as "continuous gum," by Dr. John Allen. He said, it is now one of the most æsthetic specialties, and also the best mechanical. It is not in more general use because of the lack of literature on the subject and the expense of furnace equipments, etc. Its great value lies in the fact that it so perfectly conceals the work done in conspicuous parts of the mouth. Dr. Schwartz prefers high-grade body (preferably Close's continuous gum body), because of the difficulty of getting the correct color with low fusing bodies. For crowns he prefers a porcelain veneer backed to a platinum cap. His method is given in detail for incisors and cuspids, also for bicuspids.

Porcelain bridges he makes, in most cases, with a saddle, the porcelain being baked to it. He concludes, "With the present facilities there is no reason why the progressive dentists should not do the porcelain work required in their practice.

### LINING RUBBER PLATES WITH ALUMINUM.

DR. THOS. R. PIXTON‡ gives the following method. The cast must be thoroughly hard and dry. Using the round end of

* International Den. Jour., June, 1895.

† Dental Review, May, 1895.

‡ Items of Interest, May, 1895.

a tooth-brush handle as a tool, aluminum plate, 28 guage, properly annealed, is burnished to the cast, working always from the palate toward the top of the ridge. When well-burnished down without any folds, smooth the aluminum down over the ridge, a little at a time very evenly to prevent folds; this requires practice and patience. When a good fit is secured, with a sharp enamel chisel, held at an angle of about twenty-five degrees, go all around the edges and over the palate, turning up small hooks; cut around in the opposite direction forming a double hook. This will be found quite sufficient to hold the rubber. Anneal once more to be sure that it is sufficiently soft, set up the teeth with wax, and proceed as in making an ordinary rubber plate.

Dr. Davenport, at a meeting of the New York Odontological Society,* described a method by which he had very satisfactorily made over an old rubber plate which was no longer a good fit. The piece was a full upper denture of which the arrangement of the teeth and the articulation was very satisfactory. He cut out the entire center of the plate leaving only sufficient rubber to hold the teeth together. An impression of the mouth was taken and a cast made. The teeth, as mounted on the rubber of the old plate, were placed on the cast and waxed up and finished as usual. The result was very satisfactory.

GOLD CROWNS.†

Dr. H. H. Boswell considers the habit of sticking gold crowns anywhere for a few dollars an insult to dental art. They are objectionable not only because of their detractive appearance, because of the sensitiveness of the tooth with living pulp induced by the cutting necessary to get an adaptation, and because of the cement used that mar the cervical margin, secreting fluids which decompose infecting the cement, the serum from the inflamed gum also decomposing with an odor foul beyond expression.

He advises all who do crown work to extract the tooth so as to shape and ferrule the root without overlapping margins, get-

* International Den. Jour. March, 1895.

† Cosmos, April, 1895.

ting a close metallic adaptation requiring very little cement. Use the porcelain face for all except those out of sight. Judiciously used, they are a blessing to mankind. In the hands of the unscrupulous or the unskilled, they are often a curse.

In the discussion of the subject by the Union Convention in Buffalo, Dr. W. W. Freeman spoke of the importance of the occlusion of a crown with the antagonizing natural teeth. To secure this the crown itself should be reduced with wheel or file. Grinding away the antagonizing natural tooth is apt to produce hypersensitiveness. He would not touch the natural teeth in this way under any circumstances.

Dr. A. J. Stevens* gives a few fundamental rules which should govern in placing gold crowns. The requisites are good judgment, good theory, proper tools, and mechanical and artistic skill. The natural crown should be reduced till a wire tightly twisted around the neck of the tooth will slip off without stretching. This gives the proper measure for the band. This, when soldered, should be trimmed to correspond to the gum contour and the edge beveled so that it can be burnished close to the tooth. Use 32 gage, gold plate, for the crown with cusps well reinforced. Fill the root with gutta-percha followed by gutta-percha point of proper size.

### TEMPORARY STOPPING.

Dr. J. Foster Flagg†: The present compound of red gutta-percha base plate, white wax and precipitated chalk offers the lowest grade of heat-test, and is, therefore, especially indicated in near approach to the pulp. It is almost "non-leaky" and is, therefore, valuable in protecting the pulp from medicaments or chemicals, as in sensitive dentine treatment, and in the protection of arsenical applications when not exposed to attrition. It should always be used in the pink color as a reminder of its presence. When used near the pulp it should be warmed, and used with warmed instruments. Otherwise, if properly made, warm instruments will not be found necessary.

* Items of Interest, April, 1895.
† Items of Interest, May, 1895.

THE INFLUENCE OF PREGNANCY UPON DENTAL CARIES.

"Are the teeth more liable to become carious during pregnancy?" is the question asked by R. Peterson, M.D., in a paper read before the Grand Rapids Dental Society.* Answering the affirmative, and accepting the chemico-vital theory of caries as expounded by Dr. W. D. Miller, he considers the various theories advar ced in explanation of the admitted increase of caries during pregnancy.

He fails to find any scientific foundation for the theory that the lime-salts are abstracted from the teeth to supply the demands of the fetus, the fact that the teeth are not supplied with any system of absorbents, being the most serious drawbacks to this theory; also the facts that more than sufficient lime-salts are ingested from ordinary food supplies, and that an excess of phosphates is found in the system. He attaches no value to the suggested neglect of hygienic care of the mouth, pregnancy occurring at an age when personal habits are well established, the usual bad taste in the mouth, etc., being an incentive to increased rather than lessened use of the tooth-brush, mouth-washes, etc. The most rational theory is that of changes in the character of the oral secretions, consequent upon changes in the blood and disturbance of nutrition. Increased acidity of the secretions would cause decalcification of the enamel, furnishing a more favorable soil for the development of micro-organisms. He quotes from an article by Dr. J. P. Kelly (*Journal Amer. Med. Association*), ascribing this condition of the blood to lithemia, showing a parallel, if not an identity, between the constitutional tendency produced by lithiasis and pregnancy, both originating in a grave disturbance of nutrition, preventing a similar modification of the blood, close resemblance in pathological changes, identical functional disturbances, and similar sequellæ.

The practical deduction is the indication of anti-lithemic treatment by the family physician as well as the local treatment of caries by the family dentist.

*Cosmos, March, 1895.

### GOUTY PERICEMENTITIS.

In a paper read before the Academy of Stomatology,* Dr. E. T. Darby adds to the already long list of names expressing the different phases of what was long known as Riggs' Disease. He quotes from the papers of Drs. Pierce and Black on the character and function of the alveolo-cemental membrane and the morbific influences to which it is susceptible, especially in connection with uric-acidemiar, and describes several cases of apparent abscesses on the roots of vital teeth, with gingival margin intact in the earlier attacks, pockets subsequently developing, with uremal calculus on the roots—cases difficult to account for except upon the theory of constitutional vice.

In the discussion Dr. M. L. Rhein spoke of the etiological classification of pyorrhœa alveolaris, as presented by him before the American Dental Association in the causes of pyorrhœa being as prolix as the diseases we are liable to meet with. He said that he had spent some time in visiting the medical wards of a large hospital in New York, examining the mouth of patients under treatment for various diseases, both acute and chronic, and in none of them found a healthy mucous membrane. From his observations he had reached the conclusion that we are liable to have a pyorrhœal condition follow any form of disease that will depress the nutritive action. The tissue that soonest feels the paucity of nutrition is that reached by the remotest capillaries of the circulatory system, namely: The peridental membrane and the gingival border along the roots. In the uric acid cases of abscess or pockets as described, Dr. Rhein invariably devitalizes the pulp of the afflicted teeth in view of the liability of similar deposits in the pulp itself, with the terrible neuralgic conditions met with from that cause.

Dr. Kirk said that he still has under observation the cases described at a previous meeting (see Register, page 238), and thinks that the mechanical resistance of the soft tissues determines whether the tophic abscess of gouty pericementitis shall break directly through the gum tissue and then heal up, or break

*Cosmos, and International, 1895.

along the side of the tooth leaving a fistulous track open at the gingival margin. He described a case in which tartar-lithine treatment and anti-gout regimen had arrested very rapid erosion, a case in which gold fillings on the labial surface of the anterior teeth, inserted six months previously, the cavities being the result of erosion, were "standing up like islands in the midst of the sea, the tooth-surface melted away from around them."

DR. H. H. BURCHARD ascribes the conditions to a constitutional vice leading to the retention of an excess of uric acid in the circulating fluids, which, acting as an irritant, stimulates abnormal cell-activity. The odontoblasts being thus stimulated to greater activity, the dentine becomes more dense, the pulp itself being sometimes obliterated.

Following the stimulative and the irritative stages would be the necrotic stage, necrosis, preciding the deposits. The necrotic tissues having an acid reaction the urates, insoluble in acids, are precipitated in this area.

DR. H. M. PRYOR described a case similar to one of those described by Dr. Darby.

DR. LOUIS JACK also described three cases, which, though dissimilar, illustrate the influence of gouty diathesis upon the pericemental membrane.

### CHRONIC ALVEOLAR ABSCESS WITH COMPLICATIONS.*

DR. TRUMAN W. BROPHY, in this paper, considers the various complications which are met with in cases of chronic alveolar abscess, which fail to respond to the usual treatment of cleansing the canals, and the use of antiseptics and stimulants. This may be a denuded apex from which the pericementum has been destroyed by the process of suppuration, the carious bone surrounding the apex gradually breaking down. If a superior incisor the fistula may extend into the nasal passage; if an inferior, it may extend as low as the clavicle, or even to the nipple. If a superior bicuspid or molar, the pus may find its way into the nasal passage, or it may extend into the antrum or back to the tuberosity of the maxillary bone and into the spheroidal fissure. If the pus finds

* Review, April, 1896.

its way into the antrum of Highmore, filling the cavity and closing the natural opening from the antrum to the nasal passage, there may be an elevation of the floor of the orbit, through which the pus may penetrate and escape from the corner of the eye, deceiving even a skilled ophthalmologist. Again, it may find its way into the cancellated structure of the superior maxillary bone, either making a fistulous opening in the palate or elevating the periosteum, separating it from the bone and forming a large fluctuating mass beneath this membrane. The bone, thus deprived of its periosteum, is liable to become either carious or necrosed, according to the vitality of the individual.

Another complication is that of apparent pyorrhœa alveolaris, the pus discharging at the gingival margin, being, in reality, a discharge from an alveolar abscess. This may result from partial death of the pulp, as in the buccal roots of a superior molar, the living pulp in the anterior root keeping the patient in constant pain. In this case it may respond to tests for vitality and yet have the discharge of pus from an alveolar abscess. In such cases there is the liability of accumulation of pulp nodules in the vital tissue of one root, the other root having an abscess at the apex.

In conclusion he urged the importance of careful and thorough diagnosis: "Deformities, permanent physical infirmities, septicæmia and loss of life are so frequently due to the complications of chronic alveolar abscess that a fuller comprehension of the subject should be acquired."

### THE FUNCTION OF THE PALATAL RUGÆ.

Dr. H. Burchard* attributes to the rugæ the hitherto unmentioned function of assisting the tongue in its government of the position of food during mastication, the muscles of the tongue giving a somewhat wavy motion as it engages, progressively, the succeeding rugæ, thus gathering the bolus of masticated and insalivated food into the longitudinal furrow of the tongue and squeezing it back toward the muscles of the soft palate, and the dorsum of the tongue, by which it is propelled toward the

*Cosmos, April, 1895.

pharynx—this latter being the earliest stage of deglutition mentioned by the text-books on physiology.

The fact that the lower animals also have the rugæ supports this view, making the recognized function of assisting in speech secondary to that of aiding deglutition.

SALIVARY DIGESTION.*

Recent experiments to determine the effect of saliva upon starch, under different conditions, especially the admixture of acids with other food substances show, among other results, that "oxalic acid and vinegar are so strongly inhibitory of salivary digestion that they are wholly unfit to be taken with food. The greatly less, yet distinct, action of the acids of sour fruits in hindering the action of saliva upon starch explains the reason why many persons with weak digestion are unable to take acid fruits in connection with farinaceous foods."

THE FIRST PERMANENT MOLARS—THEIR RELATION TO THE FOUR JAWS OF MAN.

A paper bearing this title, illustrated by super-imposed diagrams showing the inferior maxillary in infancy, youth, middle life and old age, by Dr. J. E. Cravens, was read before the New Jersey State Dental Society,† the paper being a plea for the retention, in the jaw, of the first permanent molar, as essential to the normal development of the anterior jaw. In the discussion of the paper Dr. B. F. Luckey would retain them only when the teeth are hard and well organized, and the denture not crowded; otherwise, he would remove all four; otherwise, with all the teeth of poor structure, and the cuspids and bicuspids crowded, the teeth at the age of fifteen or twenty will probably require filling on all the approximal surfaces. He cited the case of a young girl for whom, at the age of ten, he extracted one badly-decayed first molar, but could not gain her consent to the extraction of the other which was, therefore, filled. At the age of sixteen there was not a carious cavity on the side where the molar was extracted, while six fillings were required on the other side.

---

*Odontographic Journal, May, 1895, from Modern Medicine.

†Cosmos, April, 1895.

Mrs. Dr. White thought it possible that the badly-decayed filled first molar on that side had, perhaps, been sensitive and uncomfortable, and that the teeth on that side had decayed from not being used. She thought that if the mouth were carefully watched, and the gums promptly lanced for the eruption of the sixth-year molar, they could more frequently be saved.

Dr. J. G. Palmer thinks that extraction of these molars must result in contraction of the arch, which, if continued for two or three generations, will cause an increase rather than a decrease in irregularity. If any are extracted, it should be all four, to avoid a change in the median line.

### HYPODERMIC MEDICATION IN DENTAL PRACTICE.

Dr. W. W. Coon read a paper before the Union Convention in Buffalo* on the advantages offered by this method of medication, using the tablets as now furnished for this purpose. He suggests apomorphin as an efficient and prompt emetic; atropin to hinder pus formations, and in combination with morphin as a local anæsthetic and sedative in alveolar abscess.

Caffein for nausea or giddiness, or in case of depression following the administration of nitrous oxid; strychnin as a stimulant in the vaso-motor centers, and tannin as an antidote to strychnin; ergot in hemorrhage following extraction, also digitalis in combination with ergot; nitro-glycerin in all cases of heart-failure, hysteria, and as an antidote to cocain, etc.

### RUBEFACIENTS AND VESICANTS.

Dr. A. W. Harlan (Union Convention in Buffalo),† thinks that these remedial measures are not taken advantage of by dentists in proportion to their value. Use blisters in inflammation and rubefacients in congestion. What the patient needs is a new sensation; this he gets with a blister or a rubefacient. To obtain the desired relief from rubefaction, the rubefacient should cover ten times the inflamed area *in a locality which will draw* the blood elsewhere and relieve the tension on the arteries or arterioles. Oil of peppermint, oil of turpentine, or oil of cloves will produce a

* Cosmos, April, 1895.
† Cosmos, April, 1895.

reddening, and where used over a large area will often so alter the blood-current that there will not be anything more than swelling, without suppuration.

COAGULANTS—SELF-LIMITING.

Dr. A. W. Harlan read another paper before the Chicago Dental Society,* illustrated by new experiments, to prove his position that "Coagulating agents prevent their own diffusion through dentine where there is a coagulable material in the dentine tubes." In pulpless teeth that have contained putrescent pulps and putrescing matter for a long time the use of coagulating agents is to be avoided, as stated by Dr. Harlan, for the reason that being self-limiting they seal within the substance of the dentine the poisonous matter which ultimately escapes through the cementum and pericementum, giving a permanent lameness to the tooth, which suffers from time to time from soreness and elongation, due to imperfect sterilization of the poisoned dentine.

In the discussion Dr. E. L. Clifford said that in cases of subsequent trouble at the apical foramen he had invariably found systemic medication sufficient to relieve the pathological conditions, and had not found it necessary to remove root fillings. He thought the dentist should be sufficiently familiar with the fundamental principles of medicine to relieve these conditions. This soreness is not always an evidence of failure, but rather of nature's effort—an acute condition necessary to bring about the regeneration of the tissue, throwing out a membrane to encyst the end of the root.

Dr. Crouse said that under such conditions he should expect his patient to leave and go to some other dentist for relief. He would not know how to apologize to a patient for a swollen face after treatment of his teeth.

Dr. L. Ottofy thinks that the conditions in all such experiments are too far removed from those existing with the teeth in the human mouth to be regarded as practically conclusive. The teeth, as we treat them, are at the temperature of the body, sur-

* Review, April, 1895.

rounded by a medium that is alkaline and constantly changing as the blood-current surrounding the root of the tooth changes. The reverse of all these conditions obtains in the experiments the results of which are under discussion.

### HYDROGEN DIOXIDE.

DR. GEO. S. ALLAN gives, in "Some Notes on Hydrogen Dioxide,"* a statement of what it really is, and why it is so valuable. The affinities of oxygen are many and strong, and by its attraction for some of the elements entering into the composition of disease-germs and decomposing animal and vegetable matter, it breaks them up, forming new and harmless substances. The value of hydrogen dioxide lies in the pure, fresh oxygen it furnishes. In its pure state it is a dangerous chemical curiosity. In its available form it is held in aqueous or etherial solutions of varying strength and stability. The U. S. Pharmacopœia standard preparation is a three percent solution equivalent to ten volumes of available oxygen for one volume of solution. Brands of greater strength than this are shown not to possess good keeping qualities. "Pyrozone" three percent is shown to possess, in greater degree than any other brand in the market, freedom from excess of acid, poisonous barium salts and other impurities.

The etherial solutions, as pyrozone 5 percent, and 25 percent have the added advantage to the dentist of the aid of ether in dissolving the fatty matter encountered, and of penetrating the dental tubuli carrying with it the $H_2O_2$, the evolution of gas in the tubuli forcing the contents out leaving them in a clean, healthy condition. The 25 percent solution answers the same purpose as sodium peroxide with the advantage of being always ready for use.

Editorially† the possibilities of bleaching discolored teeth by the adaptation of the cataphoric method, using 25 percent pyrozone, is shown as follows. Let a pledget of cotton, saturated as above, be introduced into the pulp-chamber and touched with an electrode as the positive pole of a battery of low tension, the

* International, April, 1895.

† Cosmos, April, 1895.

negative electrode being either held by the patient in the hand or applied to the outer enamel surface of the tooth. By this method pyrozone is driven to ultimate ramifications of the tubuli and the oxygen is set free in direct contact with any organic debris present. The writer says this method has been tested with most satisfactory results, being in point of rapidity and efficacy far superior to applications of the same agent without the aid of the electric current.

DR. ALBERT WESTLAKE* describes this method of restoring the normal color of teeth in a few minutes, in three cases, the first experiment in this line being made March 8th. The tooth was filled immediately after bleaching, the effect on the periosteum and adjacent tissues being apparently beneficial. In the second case the application was too long, the canal filled with cement, offering greater resistance. The tooth was extremely bleached. The third case was similar to the first.

THE FAUNA OF DEAD BODIES.

According to M. Meguire† the decomposing body is reduced to dust through the aid of microbes and small insects, each stage of decay being characterized by specific organisms which are so definitely associated with successive stages of the putrefactive process that the length of time which may have elapsed since the death of the animal or person may be very accurately estimated by careful determination of the character of the microbes and insects present. This constitutes an important factor in medical jurisprudence affording additional data in determining the length of time which has elapsed since death, often an important and puzzling question.

* International, April, 1895.

† Odontographic Journal, April, 1895. (From *Modern Medicine*.)

---

A VERY small amount of alkali is sufficient to keep metal from rusting, so that if steel, iron, nickel or copper instruments are dipped in five grammes alcohol containing one or two grammes of either borate, carbonate, bicarbonate, or benzoate of soda, they will not tarnish.

